# Tracheal Stent Placement Under Remimazolam Sedation With Preserved Spontaneous Respiration: A Case Report

**DOI:** 10.7759/cureus.108989

**Published:** 2026-05-16

**Authors:** Rena Amagasa, Yumi Obata, Yu Yoshimura, Shota Yoneda, Soichiro Inoue

**Affiliations:** 1 Anesthesiology, St. Marianna University School of Medicine, Kawasaki, JPN

**Keywords:** mediastinal tumor, remimazolam, spontaneous ventilation anaesthesia, tracheal compression, tracheal stent

## Abstract

Anesthetic management for tracheal stent placement is challenging due to a shared airway and risk of hypoxemia. Propofol-based total intravenous anesthesia (TIVA) with preserved spontaneous ventilation is commonly used. Remimazolam, an ultrashort-acting benzodiazepine, may be an alternative; however, clinical experience is limited. A man in his 50s (170 cm in height, weighing 79 kg; BMI 27.3 kg/m²) with critical tracheal compression from an anterior mediastinal tumor underwent rigid bronchoscopy-guided tracheal stent placement. Anesthesia was induced with remimazolam (0.5 mg/kg/hour) and remifentanil (0.25 μg/kg/minute). Transient hypoxemia occurred twice, requiring manual ventilation and temporary interruption. The regimen was adjusted to a 7 mg remimazolam bolus followed by reduced doses (0.2 mg/kg/hour; remifentanil 0.15 μg/kg/minute), providing adequate sedation while preserving spontaneous respiration for most of the 25-minute procedure. The patient awoke promptly after flumazenil and was discharged on day 2. Remimazolam-remifentanil TIVA is feasible, but careful titration is required to avoid respiratory depression and patient movement.

## Introduction

Anesthetic management for tracheal stent placement is challenging because the airway must be shared between the anesthesiologist and the interventionist, which can lead to compromised ventilation and hypoxic events. Various anesthetic strategies have been reported, including general anesthesia with controlled ventilation using muscle relaxants, as well as techniques aimed at preserving spontaneous ventilation depending on institutional practice and patient condition [1-3]. Among these approaches, total intravenous anesthesia (TIVA) with preserved spontaneous ventilation, most commonly using a combination of propofol and opioids, has been preferred for this procedure. This approach allows stable control of anesthetic depth, whereas leakage of volatile anesthetics around the bronchoscope makes it difficult to achieve adequate inspired and alveolar concentrations with inhalational techniques [1,2].

Remimazolam is a recently introduced ultrashort-acting benzodiazepine. Continuous infusion at higher doses has been used for general anesthesia under controlled ventilation with tracheal intubation or supraglottic airway devices, whereas lower-dose infusion for gastrointestinal endoscopic procedures has recently been approved. However, reports describing the use of remimazolam in combination with an opioid for general anesthesia with preserved spontaneous ventilation remain limited in the literature [4].

In this report, we describe the case of a patient undergoing rigid bronchoscopy-guided tracheal stent placement under general anesthesia with preserved spontaneous ventilation using continuous infusion of remimazolam and remifentanil.

This article was previously presented as a meeting abstract at the 63rd Kanto-Koshinetsu and Tokyo Joint Regional Meeting of the Japanese Society of Anesthesiologists, held on September 2, 2023.

## Case presentation

**Figure 1:**
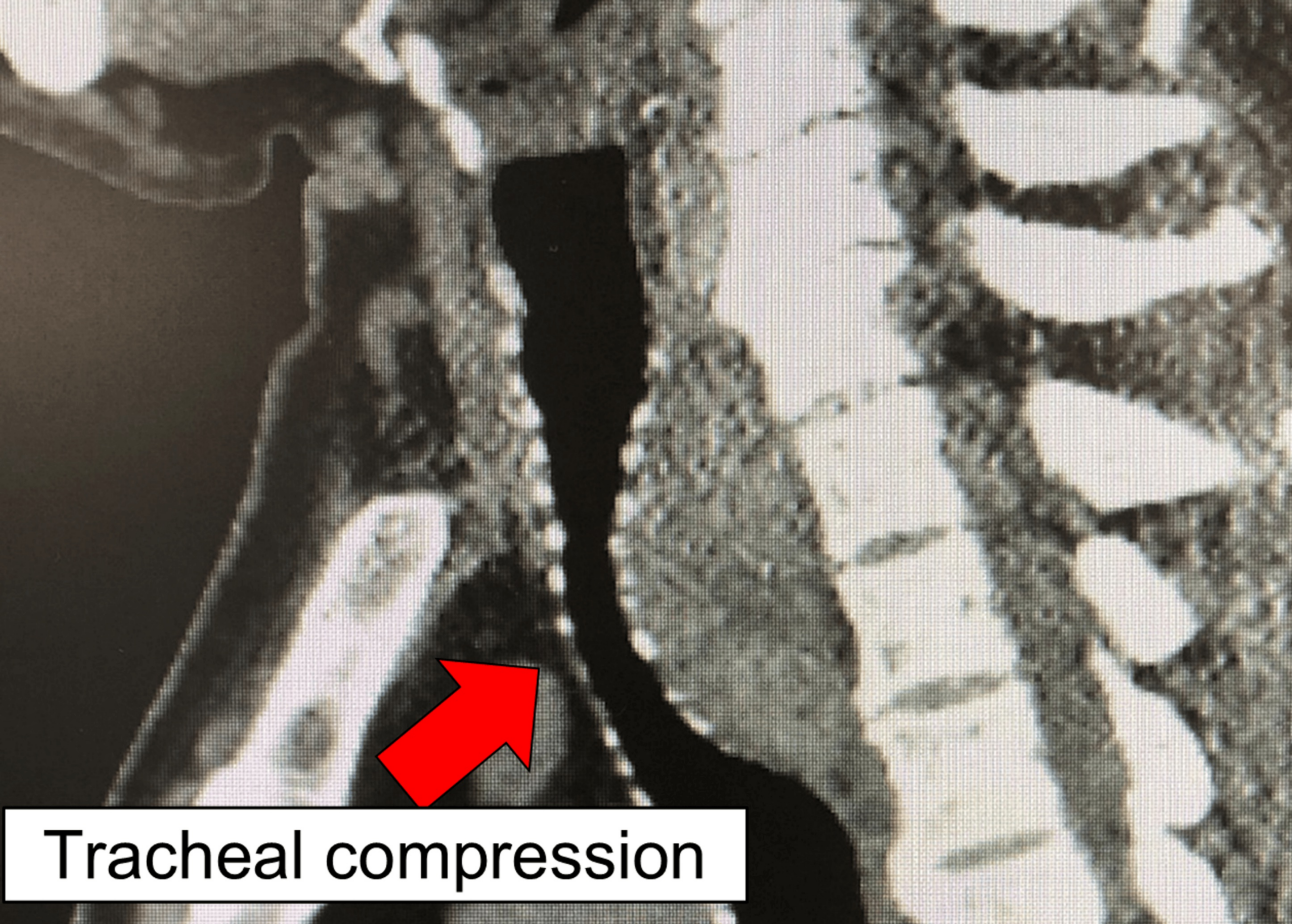
Non-contrast CT of the cervical region (sagital view) showing tracheal compression by an anterior mediastinal tumor.

A male patient in his 50s, 170 cm in height and weighing 79 kg (BMI 27.3 kg/m²), presented with dyspnea. Chest computed tomography (CT) revealed a 43 × 36 × 75 mm tumor in the upper anterior mediastinum, with tracheal compression (Figure 1). He had no significant medical history. Preoperative vital signs were blood pressure 140/87 mmHg, heart rate 78 beats/minute, and respiratory rate 16/minute. Oxygen saturation (SpO₂) was 99% on room air, despite orthopnea.

On arrival in the operating room, oxygen was administered at 6 L/minute via a face mask. Standard monitoring included electrocardiography, non-invasive blood pressure, and pulse oximetry. In addition, electroencephalographic monitoring (SedLine®; Masimo Corporation, Irvine, California, United States) and right radial arterial catheterization for invasive blood pressure monitoring were established before induction of anesthesia.

**Figure 2:**
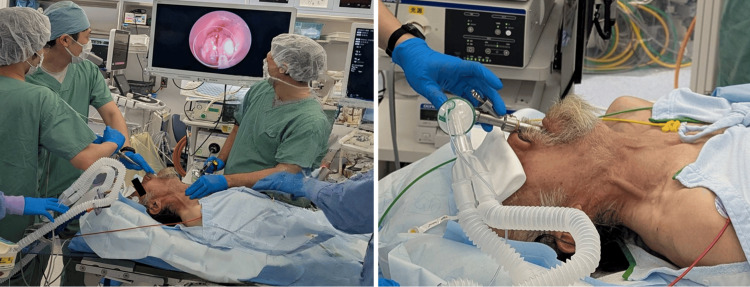
Intraoperative view during rigid bronchoscopy for tracheal stent placement, showing insertion of the rigid bronchoscope. The procedure was performed in the supine position with neck extension to achieve alignment of the airway axis. A large-diameter rigid bronchoscope (outer diameter approximately 14 mm) was inserted into the trachea and maintained in situ throughout the procedure. A self-expanding metallic tracheal stent was then deployed into the stenotic airway through the lumen of the rigid bronchoscope.

General anesthesia was induced with continuous infusion of remimazolam at 0.5 mg/kg/hour and remifentanil at 0.25 μg/kg/minute, calculated based on ideal body weight corresponding to a BMI of 22 kg/m². Approximately two minutes after initiation of the infusions, the patient lost consciousness. After induction of anesthesia, spontaneous breathing was maintained, and end-tidal CO₂ monitoring was used to assess ventilation. Topical anesthesia of the airway was sequentially performed using lidocaine applied to the oral cavity, pharynx, and larynx. During the latter part of topical airway anesthesia, before the insertion of the rigid bronchoscope, the patient’s spontaneous respiratory rate decreased, and respiratory effort became shallow, with SpO₂ decreasing to 81%. Oxygen administration was initially maintained at 6 L/minute via a face mask; however, in response to the desaturation, the oxygen flow was increased to 15 L/minute. Assisted ventilation using a face mask and breathing bag was initiated, and the infusion rates of remimazolam and remifentanil were reduced to 0.3 mg/kg/hour and 0.2 μg/kg/minute, respectively. After spontaneous respiration had sufficiently recovered and SpO₂ increased to >90%, insertion of the rigid bronchoscope was started (Figure 2).

**Figure 3:**
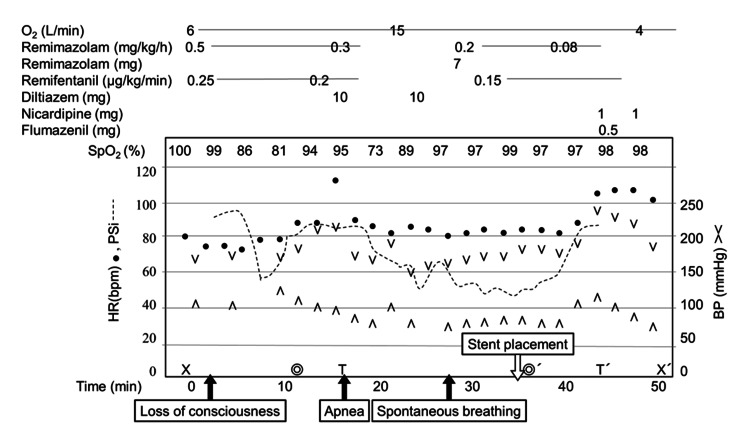
Intraoperative anesthesia record PSi: Patient State Index; BP: blood pressure; HR: heart rate; X: start of anesthesia; T: rigid bronchoscope insertion; T’: removal of the rigid bronchoscope; double circles: start and end of surgery; X’: end of anesthesia. Figure created by the authors using Microsoft PowerPoint (Microsoft Corporation, Redmond, Washington, United States).

Insertion of the rigid bronchoscope was difficult, and coughing reflexes and patient movement were observed. Approximately five minutes after scope insertion, spontaneous respiration ceased, and remimazolam and remifentanil were temporarily discontinued. Manual ventilation with O₂ at 6 L/minute through the side port of the bronchoscope resulted in a leak, and SpO₂ decreased to 73%. Subsequently, oxygen flow was increased to 15 L/minute, and oxygenation improved with manual assisted ventilation. The procedure was temporarily interrupted at this point to stabilize ventilation and oxygenation. Upon resumption of bronchoscopy, spontaneous breathing returned, and the patient became responsive. A bolus dose of remimazolam 7 mg was administered, followed by continuous infusion at 0.2 mg/kg/hour with remifentanil 0.15 μg/kg/minute. One minute later, the patient was again adequately sedated. Thereafter, deep sedation and immobility were achieved while spontaneous respiration was preserved. The tracheal stent was successfully placed with a procedure time of 25 minutes and a total anesthesia time of 52 minutes (Figure 3). Postoperatively, flumazenil 0.5 mg was administered, resulting in rapid awakening. No further respiratory depression due to re-sedation occurred. The postoperative course was uneventful, and the patient was discharged on postoperative day 2.

## Discussion

In patients with impending airway obstruction due to mediastinal tumors undergoing TIVA, preserving spontaneous ventilation may be beneficial for maintaining airway patency via upper airway muscle tone and negative intrathoracic pressure. Although general anesthesia with preserved spontaneous respiration was ultimately achieved in this case, it illustrates the inherent difficulty of maintaining stable spontaneous ventilation during rigid bronchoscopy under general anesthesia with remimazolam and remifentanil.

In this patient, respiratory instability and patient movement occurred at different phases of the procedure, including shallow breathing during the latter part of topical airway anesthesia, patient movement during bronchoscope insertion after reduction of anesthetic infusion rates, and transient apnea following bronchoscope placement. These events indicate that respiratory control was fragile and easily disrupted throughout the procedure. The observed instability was likely multifactorial, involving pharmacodynamic interaction between remimazolam and remifentanil, variations in the intensity of procedural stimulation, and the adequacy of topical airway anesthesia.

Both remimazolam and remifentanil depress respiration primarily by reducing central respiratory responsiveness to partial pressure of carbon dioxide (PaCO₂) [5], and their combination is therefore expected to produce at least additive, and possibly synergistic, respiratory depression. In a pharmacokinetic-pharmacodynamic crossover study by Vellinga et al., concomitant remifentanil administration significantly reduced the effect-site concentration of remimazolam required to achieve a comparable depth of sedation, indicating a clinically relevant pharmacodynamic interaction [6]. In the present case, respiratory compromise occurred both at a higher infusion rate during airway topical anesthesia and at a lower infusion rate after rigid bronchoscope insertion. This finding suggests that, under combined administration of these agents, the balance between anesthetic dose and the intensity of noxious stimulation may critically influence respiratory stability. Consequently, the therapeutic window between adequate suppression of patient movement to noxious stimuli and preservation of spontaneous respiration may be narrow and not readily predictable based on the dosing recommendations of either agent alone. In this case, the initial infusion rates of remimazolam and remifentanil (0.5 mg/kg/hour and 0.25 μg/kg/minute, respectively) were determined with reference to doses commonly used for general anesthesia and gastrointestinal endoscopic procedures.

However, during rigid bronchoscope insertion, coughing reflexes and patient movement were observed, followed by transient apnea. These findings suggest that the doses were insufficient to achieve immobility in response to the intense noxious stimulus of bronchoscope insertion, while approaching levels associated with respiratory depression. One possible explanation is a temporal mismatch between anesthetic and analgesic effects, in which the effect-site concentration of remifentanil may not have reached its peak at the moment of maximal procedural stimulation. In contrast, after resumption of spontaneous respiration, a reduced dosing regimen of remimazolam 0.2 mg/kg/hour and remifentanil 0.15 μg/kg/minute provided adequate anesthesia while preserving spontaneous ventilation, allowing the procedure to be completed safely. After rigid bronchoscope insertion, procedural stimulation becomes relatively constant, primarily consisting of sustained neck extension and continuous stimulation of the glottic and tracheal structures, and may be less intense than that associated with initial insertion. This change in stimulus intensity is expected to reduce anesthetic requirements. Accordingly, this latter dosing strategy may serve as a practical reference for the maintenance phase of similar procedures.

Importantly, these observations indicate that small dose changes resulted in qualitatively different clinical states, ranging from patient movement to respiratory arrest. This dose-sensitive behavior suggests the presence of a narrow and poorly defined therapeutic window under the combined administration of remimazolam and remifentanil. To better characterize this narrow therapeutic window, a systematic evaluation of the dose-response interaction between these agents, such as isobolographic analysis using respiratory depression and immobility as dual endpoints, may be warranted. To widen the narrow therapeutic window observed under combined administration of remimazolam and remifentanil, adjunctive local airway anesthesia may play a crucial role. While propofol is known to suppress pharyngeal and laryngeal reflexes [7], remimazolam appears to exert weaker reflex-suppression effects. Consequently, greater reliance on topical anesthesia is anticipated when remimazolam is used for airway procedures. Propofol-based total intravenous anesthesia has been reported to be associated with dose-dependent hypotension and respiratory depression, particularly when combined with opioids, and these effects may also be observed even at sedative doses [8,9]. Both agents are considered to exert dose-dependent effects on oxygenation, respiratory depression, and emergence; however, remimazolam may be associated with less pronounced circulatory depression even at higher doses. Nevertheless, both agents can impair ventilation depending on dose and stimulation, and careful titration is therefore essential in clinical situations requiring preservation of spontaneous ventilation. In the present case, repeated topical application of lidocaine from bronchoscope insertion through stent deployment likely attenuated airway reflexes and reduced the intensity of noxious stimulation. This may explain why immobility and preservation of spontaneous respiration were achieved at lower infusion rates during the latter half of the procedure. These findings suggest that meticulous topical airway anesthesia can effectively expand the margin between adequate immobility and respiratory compromise.

Regarding the assessment of anesthetic depth, the Patient State Index (PSi), an electroencephalographic parameter, is known to correlate to some extent with the depth of sedation [10]; however, discrepancies were observed between PSi values and the actual clinical depth of sedation. Regarding anesthetic depth assessment, although EEG-based PSi monitoring correlated to some extent with consciousness, discrepancies were noted. Spontaneous respiration ceased while PSi remained in the 80s, whereas during later surgery, PSi was maintained in the 50s with preserved spontaneous breathing. SedLine does not incorporate an algorithm specifically calibrated for remimazolam, so careful EEG and Density Spectral Array（DSA）monitoring is necessary during its use.

## Conclusions

TIVA with remimazolam and remifentanil enabled general anesthesia with preserved spontaneous ventilation for rigid bronchoscopy-guided tracheal stent placement, although transient apnea and patient movement occurred during initial dosing. These findings highlight a narrow therapeutic window between immobility and respiratory depression.
